# Suitability of bovine portion condemnations at provincially-inspected abattoirs in Ontario Canada for food animal syndromic surveillance

**DOI:** 10.1186/1746-6148-8-88

**Published:** 2012-06-22

**Authors:** Gillian D Alton, David L Pearl, Ken G Bateman, W Bruce McNab, Olaf Berke

**Affiliations:** 1 Department of Population Medicine, Ontario Veterinary College, University of Guelph, Guelph, ON, N1G 2W1, Canada; 2 Ontario Ministry of Agriculture, Food, and Rural Affairs, Guelph, ON, N1G 4Y2, Canada

## Abstract

**Background:**

Abattoir condemnations may play an important role in a food animal syndromic surveillance system. Portion condemnation data may be particularly useful, as these data can provide more specific information on health outcomes than whole carcass condemnation data. Various seasonal, secular, disease, and non-disease factors have been previously identified to be associated with whole carcass condemnation rates in Ontario provincial abattoirs; and if ignored, may bias the results of quantitative disease surveillance methods. The objective of this study was to identify various seasonal, secular, and abattoir characteristic factors that may be associated with bovine portion condemnation rates and compare how these variables may differ from previously identified factors associated with bovine whole carcass condemnation rates.

**Results:**

Data were collected from the Ontario Ministry of Agriculture, Food and Rural Affairs (OMAFRA) and the Ontario Cattlemen’s Association regarding “parasitic liver” and pneumonic lung condemnation rates for different cattle classes, abattoir compliance ratings, and the monthly sales-yard price for commodity classes from 2001-2007. To control for clustering by abattoirs, multi-level Poisson modeling was used to investigate the association between the following variables and “parasitic liver” as well as pneumonic lung condemnation rates: year, season, annual abattoir audit rating, geographic region, annual abattoir operating time, annual total number of animals processed, animal class, and commodity sales price.

**Conclusions:**

In this study, “parasitic liver” condemnation rates were associated with year, season, animal class, audit rating, and region. Pneumonic lung condemnation rates were associated with year, season, animal class, region, audit rating, number of cattle processed per year, and number of weeks abattoirs processed cattle. Unlike previous models based on whole carcass condemnations, commodity price was not associated with partial condemnations in this study. The results identified material-specific predictor variables for condemnation rates. This is important for syndromic surveillance based on abattoir data and should be modeled and controlled for during quantitative surveillance analysis on a portion specific basis.

## Background

Animal disease outbreaks can have a devastating effect, not only on animals, but to the food-animal industry, public, economy, and international trade [1-3]; therefore, research and development of novel animal disease surveillance systems is extremely important. In recent years, Ontario has experienced the emergence of various infectious animal diseases including a new strain of porcine circovirus type II (PCV-2) in 2004[4], outbreaks of bovine viral diarrhea (BVD) with enhanced virulence in cattle in the early 90’s [3], and the impact of the identification of a small number of cases of bovine spongiform encephalopathy (BSE) in Alberta, Canada in 2003[2]. The confirmation of BSE in May 2003 is just one of many examples of how the emergence of an infectious animal disease can cripple an industry and have profound lasting effects [2]. Though all BSE cases in Canada were only identified in Alberta, Canada, closure of international trade borders to Canadian cattle caused the price of cattle to drop significantly throughout the country and took several years to recover [5]. New approaches to animal disease surveillance, such as syndromic surveillance of condemnation data may be important for the timely identification of infectious animal and zoonotic disease events in the future.

Syndromic surveillance, though an increasingly popular tool in public health surveillance research [6-9] has only recently been explored as an option for animal health surveillance. One novel way to target surveillance of infectious animal and zoonotic diseases would be through the food system. In the past, abattoirs have been the focus for surveillance at this human-animal interface, usually involving targeted surveillance of a specific disease [10-13]. In recent years, research has been expanded to include surveillance of food animal data from a variety of sources including on-farm surveillance [14,15], sales-yard surveillance [16], as well as syndromic surveillance using abattoir condemnation data [17-19].

Abattoir condemnation data have the potential to provide early warning of emerging animal and zoonotic diseases, particularly provincial abattoir data and yet these data have been under-utilized in the past. Portion condemnations and whole carcass condemnation data have been previously described in the literature [20-22], however, few reports of potential usages of these data for syndromic surveillance purposes have been implemented. Changes in portion and whole carcass condemnation rates could be monitored over time and space, and when the condemnation rate reaches a certain threshold it may signal a potential outbreak or quality control problem within an abattoir and/or region. In Ontario, condemnation data from provincially inspected abattoirs are particularly useful for syndromic surveillance, as they give a relatively local perspective on the health of animals within the province. Ontario provincial abattoirs only distribute their products within the province, compared to federal abattoirs, which ship their products inter-provincially and internationally [23]. Anecdotal evidence has suggested that cattle shipped to Ontario provincial abattoirs originate from relatively local farms. This was confirmed by a previous study which found that 75% of cattle from Ontario provincial abattoirs originated from farms less than 94 kilometres from the abattoir [17]. Previous research has investigated the use of whole carcass condemnation data for syndromic surveillance [17]. It is unclear whether organ/body system data may be better suited for syndromic surveillance, as these data may provide more specific information on health outcomes than whole carcass condemnation data. By having a more specific outcome, it is hypothesized that portion condemnation data should be more sensitive than whole carcass data because inspectors condemn a carcass for one reason. However, bovine carcasses may have several disease conditions causing the condemnation of the carcass; whereas organs are less likely to have more than one reason for condemnation and reflect diseases found in a specific organ system.

Generalized linear mixed models (GLM) have been previously used for human disease surveillance [24], as well as clustered GLM’s fit by generalized estimating equations (GEE) specifically for whole carcass condemnation data [17]. Various seasonal, secular, disease, and non-disease factors were previously found to have a statistically significant association with bovine whole carcass condemnation rates, and should be controlled for in the application of quantitative surveillance methods to prevent biased results (e.g. false alarms) [17]. It was unclear whether the same factors would also be significantly associated with condemnation data when applied to portion condemnation data.

The objective of this study was to identify various seasonal, secular, and abattoir characteristic factors that may be associated with bovine portion condemnation rates and compare how these may differ from factors associated with bovine whole carcass condemnation rates. “Parasitic liver” and pneumonic lung condemnations were used in this case study as these condemnation classes were a rich source of data.

## Methods

### Data source and variables

Portion condemnation data were obtained from the Food Safety Decision Support System (FSDSS) database maintained by the Ontario Ministry of Agriculture, Food and Rural Affairs (OMAFRA). The database contains information regarding the number and reason for daily organs/body systems condemnations in provincially inspected abattoirs in Ontario. The condemnation categories of pneumonic lungs and “parasitic livers” were selected for this analysis, as these categories were among the most frequently reported portion condemnations by provincial inspectors during the study period and represent potential animal and public health concern. “Parasitic livers” is an inspection term used to label bovine livers considered unfit for human consumption, and thus condemned due to pathologies such as necrosis, fibrosis cirrhosis, atrophy, telangiectasia, and adhesions. Although the term “parasitic liver” suggests truly parasitic infections such as fascioliasis, the term covers non-parasitic conditions as well (personal communication Ab Rehmtulla, DVM, OMAFRA, Stone Road, Guelph, Ontario). Pneumonic lung condemnation refers to bovine lungs which were condemned for lesions indicative of a previous localized and resolved antero-ventral pneumonia infection. Data were extracted from the database for cattle animal classes: bulls, calves, cows, heifers, and steers from January 1, 2001 to December 31, 2007. Missing geographical coordinates for abattoirs were approximated using postal codes and/or addresses with the address geocoding software GeoPinpoint Suite 6.4 (DMTI Spatial Inc., Markham, Ontario, Canada). Using the FSDSS database, the following variables were created for each month: abattoir identification number, geographical coordinates of abattoir, year, season, number of weeks an abattoir was operating each year, total number of “parasitic liver” and pneumonic lung condemnations, total number of cattle processed each year, and animal class. Season was categorized by 3 month groupings as follows: winter (December - February), spring (March - May), summer (June - August), and fall (September - November). Animal class included five categories: bulls, cows, calves, heifers, and steers. Bulls were excluded from subsequent analyses due to missing data and inconsistencies in the use of this classification. The number of weeks an abattoir was operating each year was determined by the total number of weeks in which at least one bovine animal was processed. The total number of animals processed each year was calculated from the total number of condemned cattle plus the number of cattle fit for consumption.

Abattoir audit ratings were obtained for all abattoirs through the abattoir audit program administered through OMAFRA. The audit program assesses each facility’s food safety performance and compliance with the Ontario Meat Inspection Act. Audits are conducted once a year and evaluate each premise on 14 food safety areas based on the Standards of Compliance relating to food safety, animal welfare and occupational health and safety with a letter grade given for each abattoir [25]. Annual OMAFRA audit ratings were obtained for all abattoirs in the audit program from 2001-2007. Abattoir audit ratings were classified according to the letter grade received from best to poorest as follows: AAA, AA, A, B or C and unrated for abattoirs that had missing data.

The price of cattle was obtained from the Ontario Cattlemen’s Association market reports for 2001-2007. Prices are calculated to be the average price (in Canadian dollars) per 100 lbs based on sales records from Ontario sales-yards. A price was assigned to each month and year by animal class. The most appropriate weight category was selected to represent each animal class based on an average animal at the time of slaughter.

The agricultural region where an abattoir was located was classified as: central, eastern, northern, southern or western Ontario using the Ontario Census Agricultural Region boundaries (Statistics Canada, Census Agricultural Regions, Census year 2001). The regional location of each abattoir was determined using the point-in-polygon technique with geographic information system software ArcGIS 9.2 (ESRI, Redlands, California, USA).

Data from all sources were merged into one master dataset using Stata 10.1 (Stata Corp., College Station, Texas, USA).

### Statistical analysis

Multilevel Poisson regression modeling was used to evaluate the association of monthly condemnation rates of pneumonic lungs and “parasitic livers” with the above mentioned predictor variables. To model and evaluate their association with monthly “parasitic liver” and pneumonic lung condemnation rates, the effect of year, season, annual audit rating, number of weeks in operation, number of cattle processed, census agricultural region, animal class, sales price of animal class were included in the model. Linearity of continuous variables was assessed by plotting the log of the condemnation rate per slaughtered cattle for both liver and lung condemnations against the covariate using a locally weighted regression (lowess smoother) approach. If there was no visible linear relationship between the outcome and the covariate, and the association could not be adequately modeled with a quadratic term, or transformation, then the variable was categorized. All covariates were evaluated for statistical significance individually and then in a multivariable model using a multilevel Poisson regression model accounting for clustering of observations within abattoirs. Backward selection was based on Wald tests and non-significant covariates were removed from the model (α = 0.05). All excluded covariates were evaluated for their potential confounding effect by evaluating if their removal produced a 20% or greater change in the coefficient of the remaining variables in the model. Interactions between region and animal class, year and animal class, as well as season and animal class were investigated. The covariates included in the model were fitted using a multilevel Poisson penalized quasi-likelihood model using a 2nd order Taylor Series approximation (PQL-2). If convergence issues prohibited the multilevel Poisson model being fit using the PQL-2 algorithm, a 1st order Taylor series approximation (PQL-1) was employed. The premise’s identification number was used to account for clustering at the level of the abattoir in both models. Fit of the models were assessed visually by plotting the upper level residuals (at the level of the abattoir), also known as the best linear unbiased predictors (BLUP’s) against the normal scores to assess normality, as well as comparing the BLUP’s to the predicted outcome to assess variance homogeneity. All multi-level statistical analyses were performed using MLwiN 2.17 (Centre for Multilevel Modeling, London, UK).

## Results

### Descriptive statistics

**Figure 1:**
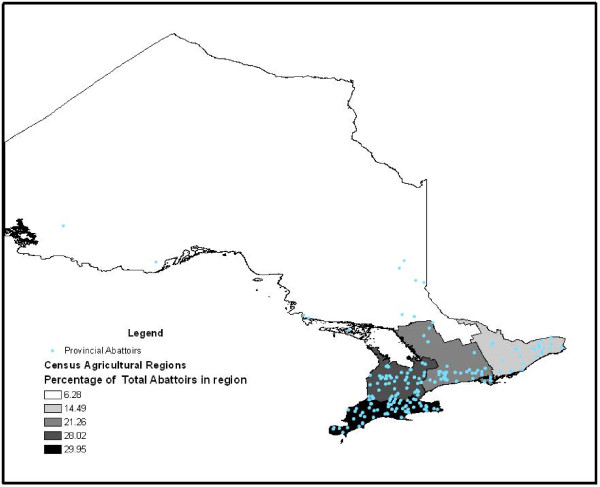
**Map of provincially-inspected abattoirs in Ontario 2001**-**2007.** Map indicating the location of provincially-inspected abattoirs processing cattle within each census agricultural region, Ontario 2001-2007

**Figure 2:**
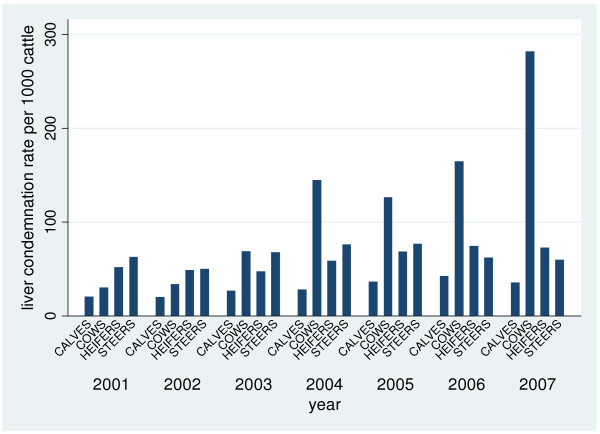
**“Parasitic liver” condemnation rates per 1000 slaughtered cattle from Ontario provincial abattoirs 2001**-**2007.** “Parasitic liver” condemnation rates per 1000 slaughtered cattle by animal class from Ontario provincial abattoirs 2001-2007

**Figure 3:**
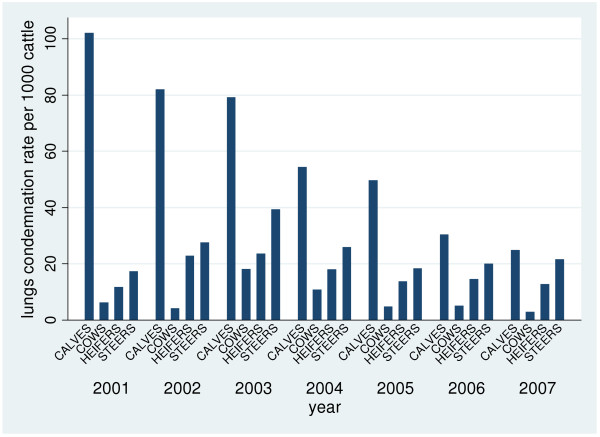
**Pneumonic lung condemnation rates per 1000 slaughtered cattle from Ontario provincial abattoirs 2001**-**2007.** Pneumonic lung condemnation rates per 1000 slaughtered cattle by animal class from Ontario provincial abattoirs 2001-2007

There were 211 provincially-inspected abattoirs slaughtering a total of 1,155,535 cattle from 2001–2007 (Figure 1). Of the total number of slaughtered cattle, 403,290 organs/portions were condemned for various reasons with the top four reasons being evidence of pneumonia, liver abscesses, nephritis and “parasitic livers”. There were 14 different organs/body systems investigated for portion condemnations and 13 condemnation reasons (Table 1). Overall, the condemnation rate for “parasitic livers” per 1000 slaughtered cattle increased over the study period, particularly in cows (Figure 2). In contrast, the condemnation rate for lungs per 1000 slaughtered cattle decreased over the study period, especially in calves (Figure 3).

**Table 1:** **Summary of Ontario provincial abattoir bovine portion condemnations 2001**-**2007**

**Organ or body system (# of condemnations/%**)	**Reason for condemnation**	**Number of portion condemnations (%)**
Front quarter	Arthritis	427 (0.11%)
(28 318/7.02%)	Inflammation	701 (0.17%)
	Abscess	955 (0.24%)
	Bruising	12 947 (3.21%)
	Contamination	13 288 (3.29%)
Head	Xanthomatosis	40 (0.01%)
(including tongue)		
(9519/2.36%)	Actino	670 (0.17%)
	Contamination	2421 (0.60%)
	Abscess	3723 (0.92%)
	Erosion	2665 (0.66%)
Heart	Adhesion	8261 (2.05%)
Hind quarter	Abscess	174 (0.04%)
Hind quarter	Inflammation	516 (0.13%)
(49 858/12.36%)	Arthritis	2132 (0.53%)
	Abscess	4349 (1.08%)
	Contamination	14 053 (3.48%)
	Bruising	28 808 (7.14%)
Kidneys	Cystic	31 570 (7.83%)
(93 589/23.21%)	Nephritis	62 019 (15.48%)
Liver	Cirrhosis	1666 (0.41%)
(168 183/41.70%)	Melanosis	8368 (2.07%)
	Adhesion	29 125 (7.22%)
	Abscess	57 511 (14.26%)
	Parasitic	71 513 (17.73%)
Loin	Abscess	456 (0.11%)
Ribs	Abscess	46 (0.01%)
Shoulder/limb	Arthritis	214 (0.05%)
Stifle joint	Arthritis	693 (0.17%)
Trim	Bruising	7096 (1.76%)
Lungs	Pneumonia	36 883 (9.15%)
Total portions condemned		403 290
Total cattle slaughtered		1 155 535

Total number of cattle organs/body system condemned and reason for condemnation in provincially inspected abattoirs in Ontario, 2001-2007.

The quartiles of total number of animals and the corresponding number of processing abattoirs were tabulated for each year of the study period (Table 2a). With the exception of 2004 and 2005, most of the abattoirs processed fewer than 500 cattle per year. The quartiles of the total number of weeks each year an abattoir processed at least one animal and the corresponding number of abattoirs was tabulated for each year of the study period (Table 2b). Over the study period, there was an increasing trend in the number of abattoirs processing cattle more than 49 weeks per year. The annual OMAFRA audit rating scores and the corresponding number of abattoirs receiving those scores are shown in Table 2c for each year of the study. Throughout the study period, the majority of rated abattoirs were given an “A” rating. The median sales price of each animal class was calculated for each month during the study period (Table 2d). The median sales-prices for all cattle classes were lowest in 2004. No continuous variables were found to have a linear relationship with cattle carcass condemnation rates, therefore, quartiles were used to categorize the total number of animals processed and number of weeks an abattoir was open (Tables 2a and 2b, respectively). Price was categorized into a dichotomous variable according to whether the price was less than or equal to the annual median sales price for each animal class (Table 2d).

**Table 2:** Summary of number of cattle processed, number weeks open, audit rating and animal class

**a) Number of animals processed**	**Number of Abattoirs**						
	**2001**	**2002**	**2003**	**2004**	**2005**	**2006**	**2007**
1-286	73	72	36	24	30	40	38
287-498	43	35	40	28	36	34	26
499-841	24	24	36	40	40	32	35
842-27 286	22	26	33	49	41	32	30
b) Number of weeks open	Number of Abattoirs						
1-43	66	67	44	32	43	38	38
44-49	56	45	48	45	30	39	29
50-51	18	28	25	31	41	31	32
52	22	17	28	33	33	30	30
c) Audit Rating	Number of Abattoirs						
AAA	1	0	0	0	0	1	1
AA	6	6	7	13	15	22	28
A	61	71	77	76	78	78	72
B	14	8	8	5	8	6	9
C	2	1	0	0	1	0	0
Unrated	78	71	53	47	45	31	19
d) Animal Class	^1^Median sales-price of animal class per year 2001-2007						
Calves	136.53	117.95	112.19	83.61	107.14	119.69	108.49
Cows	64.28	58.64	27.11	19.70	25.38	31.84	34.77
Heifers	112.01	101.22	82.16	73.61	87.74	90.87	91.09
Steers	113.24	102.57	83.15	75.60	91.07	93.55	92.56

^1^Median sales-price in Canadian dollars per 100 lbs.

Summary of the a) quartiles of the number of cattle processed, b) quartiles of the number of weeks at least one bovine animal was processed, c) annual OMAFRA audit rating, and d) median sales price of calves, cows, heifers, steers 2001–2007 for portion condemnations.

### Statistical models

#### Pneumonic lung condemnation model

Results of the univariable Poisson regression models (PQL-2) indicated that animal class (p < 0.01), year (p < 0.01), season (p < 0.01), agricultural region (p = 0.02), number of weeks abattoirs processed cattle (p < 0.01) all had statistically significant association with the condemnation rate of lungs according to the Wald test for the variable. The number of animals processed per year (p < 0.01) was statistically significant according to the Wald test for the variable using a MQL estimation method, as the PQL algorithm would not converge. Price was not significantly associated with lung condemnations (p = 0.79). Due to the convergence issues in the model fitting process the univariable association between the outcome and audit rating could not be assessed.

**Figure 4:**
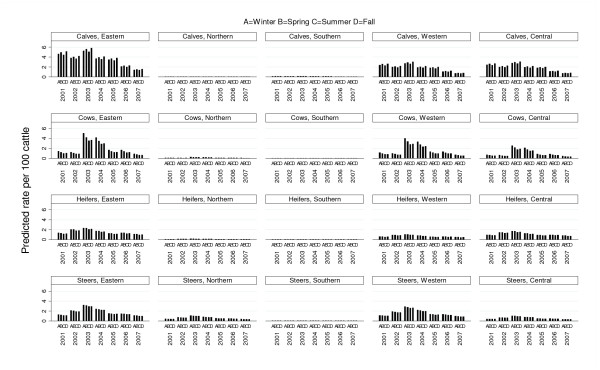
**Model expected pneumonic lung condemnation rates based on multi-level Poisson model.** Model expected pneumonic lung condemnation rates estimated using results of the multivariable multi-level Poisson regression model based on data from Ontario provincial abattoirs (2001-2007)

Animal class, year, season, agricultural region, audit rating, number of weeks abattoirs processed cattle, and number of animal processed per year were found to have a statistically significant association with the outcome in the multivariable model (PQL-2) (Table 3). There was no evidence that the excluded variable price confounded these variables. Statistically significant interactions were found to exist between animal class and season, animal class and region, as well as, animal class and year. The fitted model indicated that lung condemnations tended to be lower in higher rated abattoirs compared to C rated abattoirs (Table 3). Condemnation rates tended to be lower in abattoirs processing a larger number of cattle each year compared to smaller processing abattoirs (Table 3). Lung condemnation rates were higher in abattoirs open throughout the year compared to abattoirs open fewer weeks during the year (Table 3). Due to the complexity of the interaction terms between animal class and season, agricultural region and year, relationships among these variables based on the predicted rates from the fitted model were explored (Figure 4). According to predicted lung condemnation rates for calves from the multilevel model, condemnation rates were highest in eastern, western and central Ontario regions (Figure 4), with the highest condemnation rates found in calves in eastern Ontario compared to all other regions and animal classes. A decreasing trend in condemnation rates in calves was also seen in these same regions with the exception of a small peak in 2003. The same decreasing trend and peak in 2003 was also evident in heifers and steers in the same regions noted above (Figure 4). The same trend was evident in cows, with the exception of a peak in 2003 and 2004 (Figure 4). In comparison, calves, cows, heifers and steers in northern and southern Ontario regions had consistently lower lung condemnation rates throughout the entire study period (Figure 4). The best linear unbiased predictors (BLUP’s) were visually inspected for the multi-level model; there was no evidence to reject the assumptions of normally distributed residuals and homogeneity of variance.

**Table 3:** Multivariable multi-level Poisson regression using pneumonic lung condemnation rates

**Variable**	**Categories**	**IRR1**	**Std. Err.**	**P-value**		**95% CI**
Year	2001	—	—	—	—	—
	2002	0.83	0.02	< 0.01	0.79	0.86
	2003	1.14	0.02	< 0.01	1.09	1.19
	2004	0.80	0.03	< 0.01	0.76	0.84
	2005	0.75	0.03	< 0.01	0.71	0.79
	2006	0.45	0.03	< 0.01	0.43	0.49
	2007	0.31	0.04	< 0.01	0.28	0.34
Animal class	Calves	—	—	—	—	—
	Cows	0.30	0.23	< 0.01	0.19	0.47
	Heifers	0.39	0.11	< 0.01	0.32	0.49
	Steers	0.16	0.07	< 0.01	0.14	0.19
Season	Winter	—	—	—	—	—
	Spring	1.07	0.02	< 0.01	1.03	1.11
	Summer	0.96	0.02	0.03	0.93	0.99
	Fall	1.10	0.02	< 0.01	1.06	1.15
Region	Central	—	—	—	—	—
	Eastern	1.88	0.54	0.24	0.66	5.39
	Northern	0.02	1.31	< 0.01	0.001	0.20
	Southern	0.75	0.47	0.54	0.30	1.90
	Western	0.98	0.46	0.97	0.40	2.42
Rating	C	—	—	—	—	—
	AAA	0.24	1.26	0.26	0.02	2.85
	AA	0.18	0.64	0.01	0.05	0.62
	A	0.11	0.63	< 0.01	0.03	0.37
	B	0.15	0.63	< 0.01	0.04	0.50
	unrated	0.29	0.64	0.048	0.08	0.99
# of animals	1–286	—	—	—	—	—
	287–498	0.70	0.12	< 0.01	0.56	0.89
	499–841	0.36	0.15	< 0.01	0.27	0.48
	842–27286	0.27	0.14	< 0.01	0.20	0.35
# of weeks	1–43	—	—	—	—	—
	44–49	1.72	0.07	< 0.01	1.50	1.97
	50–51	2.84	0.06	< 0.01	2.55	3.16
	52	2.31	0.05	< 0.01	2.11	22.54
Season x animal class	Winter x calves	—	—	—	—	—
	Spring x heifers	0.93	0.05	0.14	0.85	1.02
	Summer x heifers	0.92	0.05	0.10	0.83	1.02
	Fall x heifers	0.83	0.05	< 0.01	0.76	0.92
	Spring x steers	0.91	0.04	0.01	0.84	0.98
	Summer x steers	0.94	0.04	0.10	0.88	1.01
	Fall x steers	0.82	0.04	< 0.01	0.77	0.88
	Spring x cows	0.78	0.09	0.01	0.66	0.93
	Summer x cows	0.73	0.09	< 0.01	0.61	0.88
	Fall x cows	0.65	0.09	< 0.01	0.55	0.78
Region x animal class	Central x calves	—	—	—	—	—
	South x heifers	0.37	0.09	< 0.01	0.31	0.45
	West x heifers	0.65	0.09	< 0.01	0.53	0.78
	North x heifers	6.58	1.25	0.13	0.56	76.70
	East x heifers	0.72	0.16	0.05	0.54	0.99
	South x steers	0.68	0.07	< 0.01	0.60	0.78
	West x steers	2.81	0.06	< 0.01	2.52	3.14
	North x steers	67.90	1.06	< 0.01	8.44	546.45
	East x steers	1.64	0.14	< 0.01	1.25	2.15
	South x cows	0.26	0.27	< 0.01	0.15	0.45
	West x cows	1.60	0.20	0.02	1.09	2.34
	North x cows	7.39	1.49	0.18	0.40	136.25
	East x cows	1.05	0.24	0.85	0.66	1.66
Year x animal class	2001 x calves	—	—	—	—	—
	2002 x heifers	1.84	0.08	< 0.01	1.59	2.13
	2003 x heifers	1.53	0.08	< 0.01	1.32	1.78
	2004 x heifers	1.64	0.08	< 0.01	1.41	1.92
	2005 x heifers	1.24	0.08	< 0.01	1.06	1.45
	2006 x heifers	2.23	0.08	< 0.01	1.90	2.63
	2007 x heifers	2.68	0.09	< 0.01	2.25	3.20
	2002 x steers	2.05	0.05	< 0.01	1.85	2.27
	2003 x steers	2.27	0.05	< 0.01	2.06	2.49
	2004 x steers	2.44	0.05	< 0.01	2.20	2.69
	2005 x steers	1.64	0.05	< 0.01	1.48	1.83
	2006 x steers	2.60	0.06	< 0.01	2.32	2.92
	2007 x steers	2.86	0.06	< 0.01	2.54	3.23
	2002 x cows	1.07	0.19	0.74	0.73	1.55
	2003 x cows	3.09	0.14	< 0.01	2.35	4.08
	2004 x cows	3.63	0.14	< 0.01	2.76	4.77
	2005 x cows	1.57	0.15	< 0.01	1.17	2.12
	2006 x cows	2.57	0.16	< 0.01	1.89	3.49
	2007 x cows	2.08	0.18	< 0.01	1.46	2.98

^1^Incidence rate ratio.

Multivariable multi-level Poisson regression model (PQL-2) investigating the association between pneumonic lung condemnation rates in Ontario provincial abattoirs and year-animal class interaction, season-animal class interaction, region-animal class interaction, number of animals, number of weeks of processing and audit rating.

#### “*Parasitic liver*” condemnation model

Results of the univariable multi-level Poisson regression model (PQL-2) indicated that animal class (p < 0.01), year (p < 0.01), season (p < 0.01), agricultural region (p < 0.01), audit rating (p < 0.01), the number of weeks an abattoir processed cattle (p < 0.01), total number of cattle processed per year (p < 0.01) and price (p < 0.01) all had statistically significant associations with “parasitic liver” condemnation rate according to the Wald test for the variable.

**Figure 5:**
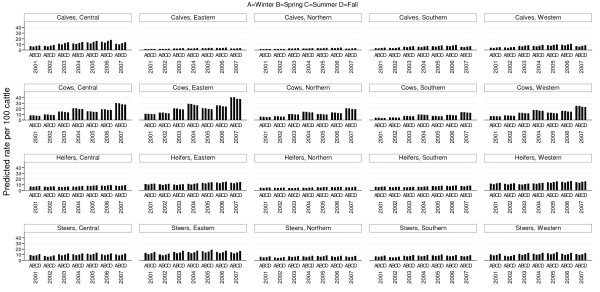
**Model expected “parasitic liver” condemnation rates based on multi-level Poisson model.** Model expected “parasitic liver” condemnation rates estimated using results of the multivariable multi-level Poisson regression model based on data from Ontario provincial abattoirs (2001-2007)

In the multivariable multilevel Poisson model (PQL-1), year, animal class, season, agricultural region, and audit rating were found to have a statistically significant association with the outcome (Table 4). There was no evidence that the excluded variables confounded the variables included in the model. Statistically significant interactions were found to exist between animal class and season, animal class and region, as well as animal class and year (Table 4). The fitted model indicated that liver condemnation rates were higher in C rated abattoirs compared to higher rated abattoirs (Table 4). Due to the complexity of the interaction terms between animal class and season, agricultural region and year, relationships among these variables based on predicted rates from the fitted model were explored (Figure 5). According to predicted liver condemnation rates for steers from the multilevel model, condemnation rates were highest in eastern Ontario. Rates amongst steers tended to be higher in fall and winter. The lowest rates in steers were found in northern Ontario (Figure 5). Liver condemnation rates for calves were highest in central Ontario and lowest in eastern and northern Ontario. Similar to steers, rates amongst calves tended to be higher in fall and winter throughout the study period. Liver condemnation rates in cows had the highest rates compared to all other animal classes in all regions of Ontario throughout the study period (Figure 5). Condemnation rates in cows were highest in central and eastern Ontario and lowest in southern Ontario. Condemnation rates amongst cows remained fairly stable throughout the seasons; however, an increasing secular trend was seen in cows with peaks in 2004 and 2007 for all regions of Ontario. Liver condemnation rates for heifers were highest in eastern and western Ontario and lowest in northern and southern Ontario (Figure 5). Condemnation rates in heifers remained fairly stable within a region throughout the study period. The predicted random effects were subjected to a visual diagnostic analysis and no evidence against the assumption of normality and variance homogeneity was found.

**Table 4:** Multivariable multi-level Poisson regression using “parasitic liver” condemnation rates

**Variable**	**Categories**	**IRR1**	**Std. Err.**	**P-value**		**95% CI**
Year	2001	—	—	—	—	—
	2002	1.08	0.04	0.08	0.99	1.17
	2003	1.73	0.04	< 0.01	1.60	1.88
	2004	1.81	0.04	< 0.01	1.67	1.96
	2005	2.06	0.04	< 0.01	1.90	2.24
	2006	2.29	0.04	< 0.01	2.12	2.49
	2007	1.71	0.04	< 0.01	1.57	1.86
Animal class	Calves	—	—	—	—	—
	Cows	1.00	0.06	1.00	0.89	1.13
	Heifers	1.45	0.05	< 0.01	1.30	1.60
	Steers	1.29	0.08	< 0.01	1.10	1.50
Season	Winter	—	—	—	—	—
	Spring	0.88	0.03	< 0.01	0.83	0.94
	Summer	1.08	0.03	< 0.01	1.02	1.14
	Fall	1.22	0.03	< 0.01	1.15	1.29
Region	Central	—	—	—	—	—
	Southern	0.49	0.18	< 0.01	0.35	0.69
	Western	0.61	0.18	< 0.01	0.43	0.86
	Northern	0.22	0.31	< 0.01	0.12	0.41
	Eastern	0.23	0.22	< 0.01	0.15	0.35
Rating	C	—	—	—	—	—
	AAA	0.53	0.13	< 0.01	0.41	0.69
	AA	0.72	0.09	< 0.01	0.60	0.85
	A	0.72	0.09	< 0.01	0.60	0.85
	B	0.74	0.09	< 0.01	0.62	0.88
	unrated	0.58	0.10	< 0.01	0.48	0.71
Season x animal class	Winter x calves	—	—	—	—	—
	Spring x heifers	1.04	0.04	0.37	0.96	1.12
	Summer x heifers	0.96	0.04	0.33	0.90	1.04
	Fall x heifers	0.90	0.04	0.00	0.84	0.97
	Spring x steers	0.96	0.04	0.30	0.90	1.03
	Summer x steers	0.89	0.03	< 0.01	0.84	0.95
	Fall x steers	0.95	0.03	0.11	0.89	1.01
	Spring x cows	1.13	0.04	< 0.01	1.05	1.21
	Summer x cows	0.87	0.04	< 0.01	0.81	0.93
	Fall x cows	0.75	0.03	< 0.01	0.70	0.80
Region x animal class	Central x calves	—	—	—	—	—
	South x heifers	1.90	0.05	< 0.01	1.71	2.10
	West x heifers	1.82	0.05	< 0.01	1.65	2.00
	North x heifers	2.96	0.17	< 0.01	2.11	4.15
	East x heifers	7.34	0.09	< 0.01	6.10	8.82
	South x steers	1.55	0.05	< 0.01	1.42	1.69
	West x steers	1.76	0.04	< 0.01	1.63	1.91
	North x steers	2.90	0.17	< 0.01	2.09	4.01
	East x steers	6.09	0.09	< 0.01	5.10	7.28
	South x cows	0.96	0.06	0.45	0.85	1.07
	West x cows	1.38	0.05	< 0.01	1.26	1.57
	North x cows	3.12	0.17	< 0.01	2.24	4.34
	East x cows	5.96	0.09	< 0.01	4.96	7.17
Year x animal class	2001 x calves	—	—	—	—	—
	2002 x heifers	0.88	0.06	0.02	0.79	0.98
	2003 x heifers	0.52	0.06	< 0.01	0.47	0.58
	2004 x heifers	0.57	0.05	< 0.01	0.51	0.63
	2005 x heifers	0.58	0.05	< 0.01	0.53	0.65
	2006 x heifers	0.55	0.05	< 0.01	0.50	0.61
	2007 x heifers	0.72	0.05	< 0.01	0.65	0.80
	2002 x steers	0.74	0.05	< 0.01	0.67	0.82
	2003 x steers	0.65	0.05	< 0.01	0.59	0.72
	2004 x steers	0.63	0.05	< 0.01	0.58	0.69
	2005 x steers	0.60	0.05	< 0.01	0.55	0.66
	2006 x steers	0.50	0.05	< 0.01	0.45	0.54
	2007 x steers	0.64	0.05	< 0.01	0.59	0.71
	2002 x cows	1.09	0.09	0.32	0.92	1.29
	2003 x cows	1.06	0.08	0.46	0.91	1.23
	2004 x cows	1.41	0.07	< 0.01	1.23	1.63
	2005 x cows	0.91	0.07	0.17	0.79	1.04
	2006 x cows	1.01	0.07	0.88	0.88	1.16
	2007 x cows	2.12	0.07	< 0.01	1.83	2.44

^1^Incidence rate ratio.

Multivariable multi-level Poisson regression model (PQL-1) investigating the association between “parasitic liver” condemnation rates in Ontario provincial abattoirs and year-animal class interaction, season-animal class interaction, region-animal class interaction and audit rating.

## Discussion

Portion and whole carcass condemnation data may have an important role in the development of a food animal syndromic surveillance system. These data provide insight into lesions on carcasses and organs, which may lead to early detection of emerging animal and zoonotic diseases. This study builds upon previous research investigating biological and non-biological factors associated with bovine whole carcass condemnation rates in Ontario provincial abattoirs during the same study period [17]. As with whole carcass data, various seasonal, secular and abattoir characteristic factors were found to have an association with liver and lung portion condemnations, and need to be taken into account in the application of quantitative methods, such as cluster detection for disease surveillance involving these data. In addition, the results show differences in the models constructed for liver and lung portion condemnations, as well as between portion and previously explored whole carcass condemnation data [17]. These findings suggest that different variables may be associated with condemnation rates depending on the type of material being condemned, and should be modeled and controlled for during quantitative surveillance on a portion-specific basis. Previous studies have demonstrated the importance of identification of potential confounding variables and different methods of controlling for these variables in cluster detection methods for disease surveillance [26,27]. For example, a study by Kleinman et al. [26] compared the performance of the space-time scan statistic using unadjusted and covariate-adjusted respiratory complaint data in humans to account for confounding temporal factors such as day of the week, month and holidays. The study concluded that failure to adjust for confounding variables can produce many false alarms and/or mask potential outbreaks.

Pneumonic lungs and “parasitic” livers were used as examples to explore the modeling of biological and non-biological factors associated with portion condemnations. These condemnation designations were selected as they represented two of the most frequently reported reasons for portion condemnations by inspectors at provincial abattoirs during the study period. Syndromic surveillance is based on non-traditional data sources. Though this allows for early warning about potential disease outbreaks these systems are generally less sensitive than traditional laboratory based surveillance systems [28]. Therefore it is of utmost importance to (i) use highly predictive models (adjusted for known risk factors and confounders), and (ii) preserve the robustness of the models by adhering to the principle of parsimony. These are well known contradicting goals in predictive modeling that make sufficient/large sample sizes an important requirement. Therefore, pneumonic lung and “parasitic liver” condemnation rates were selected as they were a rich data source from which to estimate trends. Livers are important from a public health and economic standpoint, as they are a common edible portion in cattle and represent a possible food safety concern. Lungs, though generally not consumed by the average Ontarion, are also an important animal and public health concern, as lesions such as tuberculosis granulomas may be found in inedible organs/tissues such as the lungs. These are important factors to consider when selecting portion condemnation designations for syndromic surveillance; a previous study by Thomas et al. [19], investigating the use of portion condemnations in market hogs, noted that the quality of data recording was poor for organs that were not considered to be economically important or a concern for food safety.

It was interesting to find similarities and differences in terms of the significant variables and the impact the variables had on condemnation rates in both the liver and lung portion models, as well as the previously described models for whole carcass condemnations [17]. The variables year, animal class, season and annual audit rating were found to be significantly associated with condemnation rates in both portion models as well as whole carcasses. It is not surprising that season and animal class were found to be significant factors associated with abattoir condemnation rates in all three models, as many animal diseases tend to have a distinct seasonality and high risk age groups associated with the disease. For example, bovine respiratory disease complex more commonly infects calves following a stressful event, such as sudden change in weather conditions [29], and older cattle are generally at higher risk for being culled due to disease and thus condemned more frequently. In the whole carcass condemnation data, the variable year, identified patterns assumed to be associated with the discovery of Bovine Spongiform Encephalopathy (BSE) in Alberta, Canada in 2003 [17]. In the portion models, year also appeared to have a significant decreasing trend in calves for pneumonic lung condemnations. It is suspected that these temporal trends in liver and lung condemnations also stem from regulation changes due to BSE. Prior to BSE in Canada, it was legal to load and transport downer animals with a veterinary certification of fitness for slaughter. However, during 2004, changes were made to federal cattle transportation regulations which forbid the transportation and slaughter of non-ambulatory animals [30]. Ambulatory animals may be less likely to have lung pathology than compromised and downer animals and are likely reflected in the marked decrease in condemnation rates in pneumonic lungs (personal communication Ab Rehmtulla, DVM, OMAFRA, Stone Road, Guelph, Ontario). In contrast, “parasitic liver” condemnations increased in cows over the study period and may also reflect the type and quality of cattle being sent to slaughter; however, it is unclear why an increase was seen over the study period. Audit rating appeared to be an important variable for provincial abattoir condemnation data. It was hypothesized that audit rating may reflect an abattoir’s compliance to regulations and/or willingness to accept animals of poorer quality. Although, different trends were found in these variables between all 3 models, the overall statistically significant association of these variables with condemnation rates appears to be “universal” within Ontario provincial abattoir data, and should be controlled for in quantitative cluster detection methods for surveillance. Although the AAA and C categories represent a small number of abattoirs over the study period, we felt that it was important to not collapse the categories, as these abattoirs point to unique qualities among these establishments. Since this study was conducted, the audit rating system was simplified in September 2010 to a 3-grade system including pass, conditional pass and fail to better reflect systems used in several jurisdictions for evaluating food safety at restaurants [25]. This change in reporting may need to be accounted for in future studies and/or in attempts to control for non-disease issues when conducting space-time cluster analyses.

Agricultural region was found to be significantly associated with the portion condemnation rates but not with whole carcass rates. It was interesting to note that overall, predicted condemnation rates for bovine lungs and liver were lower in northern and southern Ontario regions compared to other regions for all animal classes throughout the study period. This pattern was particularly evident in the lung dataset. This regional difference of condemnation rates in pneumonic lungs may reflect a genuine regional difference; however, it likely stems from the quality of animals being sent to slaughter in these regions. Abattoirs in southern and northern Ontario did not specialize and rarely received non-ambulatory cattle. Prior to 2004, most of the so-called “downer plants” were located in southwestern, central and eastern Ontario. While the regional differences in “parasitic” livers may be due to a smaller concentration of dairy cattle in northern and to some extent southern Ontario, corresponding with the lower condemnation rates in these areas; it was surprising that variables related to abattoir processing capacity, such as number of cattle processed each year and number of weeks an abattoir processed cattle was only significant in the lung condemnation model. This may reflect issues with processing speeds within abattoirs, perhaps the speeds impact lung inspection more than livers since livers are more commonly considered to be an edible portion of cattle and thus more carefully inspected.

It is important to identify and understand the factors which may cause “noise” in the data before any quantitative methods can be chosen for disease surveillance. This extra work beforehand can save valuable time and resources investigating “false alarms” after the application of quantitative methods. Although differing results were found in the two portion models as well as the previously described whole carcass model, common themes arose from the results. Bovine abattoir condemnation data are sensitive to the effects of regulatory and economic changes in the industry. Therefore it is important to adjust models as regulations change over time. In addition, seasonal, secular, and non- disease factors, such as commodity class, abattoir rating and processing capacity also seemed to be important factors for bovine abattoir condemnation data and should be adjusted in subsequent cluster detection analyses to prevent biased results. All analyses thus far have been conducted retrospectively, facilitating the use of historical data to highlight variables which need to be taken into account prior to applying outbreak detection methods. However, the practical application of disease surveillance would be conducted prospectively, and would have implications on the models. For example, the variable year was found to be a very important variable in all of the models; however this variable would not be applicable in a prospective analysis. The secular trend effect would have to be accounted for in the analyses in some other way, for example using a trend polynomial or trend filter. In addition, we were only able to account for clustering by abattoir. However, it would be useful to explore the effect of clustering by inspector as well. Unfortunately, these data were not available to explore the effect of inspector. Specific training of inspectors may also improve the ability of a syndromic surveillance system to detect unusual events. For example, the broad use of the category “parasitic liver” may be more useful at detecting changes in disease if inspectors used more specific criteria when condemning organs.

The suitability of portion vs. whole carcass condemnation data for syndromic surveillance is difficult to ascertain in this study. It is suspected that for syndromic surveillance purposes, portion condemnation data will be more specific and sensitive at detecting changes in condemnation rates. However, validation of this conclusion is difficult with no major documented disease outbreaks in Ontario cattle during the study period. Nevertheless, it is encouraging that regulatory changes surrounding the identification of BSE in Alberta, Canada was identified in both the whole carcass and portion condemnation data. A similar study in pigs using Ontario provincial abattoir data found that whole hog carcass condemnation data performed better than portion carcass condemnation data at detecting disease clusters consistent with a documented porcine circovirus-associated disease outbreak in Ontario [19]. Further investigations, perhaps using simulated data, are needed to determine which types of data are more suitable for the syndromic surveillance of specific types of diseases.

## Conclusions

Findings from this study suggest that there are “universal” factors associated with condemnation data, such as animal class, year, season and audit rating, additional material-specific covariates are important and should be modeled and controlled for in quantitative methods for disease surveillance, such as in cluster detection methods. Validation of results is an issue with bovine portion abattoir condemnation data, as there were no documented outbreaks in Ontario cattle during the study period. Further investigations are needed to determine whether portion data would perform better than whole carcass condemnation data for disease surveillance, and whether certain portions are more appropriate than whole carcass for the surveillance of specific diseases.

## Authors’ contributions

GDA performed the statistical analysis and drafted the manuscript. WBM was involved in the acquisition of data, and the drafting and revising the manuscript for intellectual content. DLP and OB were involved in the conception and design, analysis and interpretation of data, and revising manuscript critically for important intellectual content. KGB was involved in the interpretation of data, and the revising of the manuscript critically for important intellectual content. All authors read and approved the final manuscript.
